# The first Nearctic record of the genus *Neocheiridium* (Pseudoscorpiones: Cheiridiidae), with description of *Neocheiridium gullahorum* sp. n.

**DOI:** 10.3897/BDJ.8.e48278

**Published:** 2020-01-24

**Authors:** Kaarel Sammet, Olavi Kurina, Hans Klompen

**Affiliations:** 1 Estonian University of Life Sciences, Institute of Agricultural and Environmental Sciences, Tartu, Estonia Estonian University of Life Sciences, Institute of Agricultural and Environmental Sciences Tartu Estonia; 2 The Ohio State University, Museum of Biological Diversity, Columbus, Ohio, United States of America The Ohio State University, Museum of Biological Diversity Columbus, Ohio United States of America

## Abstract

**Background:**

The genus *Neocheiridium* Beier, 1932 currently contains seven Neotropical (including Caribbean) and two Afrotropical species, with no Nearctic records.

**New information:**

An undescribed species of *Neocheiridium* from South Carolina was discovered in the Ohio State University Acarology Collection and is described as *Neocheiridium gullahorum*, n. sp. (based on specimens of both sexes). A key to known world species of *Neocheiridium* is proposed.

## Introduction

**Figure 1.**
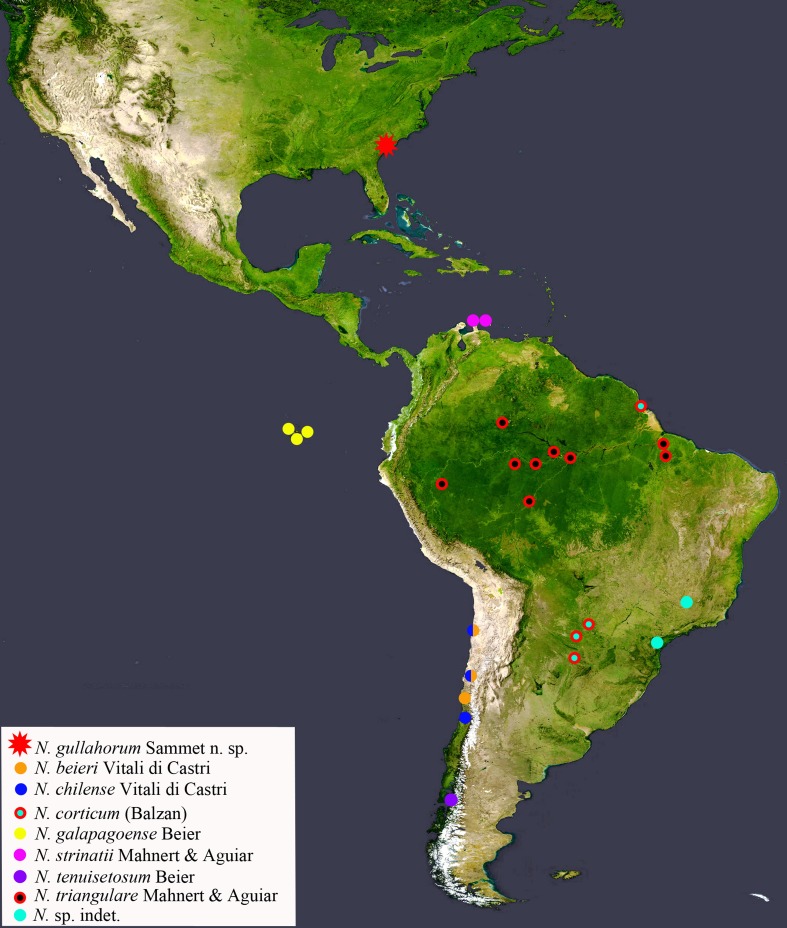
Currently known distribution of *Neocheiridium* species in the Americas. Some symbols indicate multiple nearby finding localities (based on Aguiar and Bührnheim 1998, Beier 1959, Beier 1978, Harvey 2013, Mahnert 2014, Mahnert and Aguiar 1986, Vitali-di Castri 1962, Von Schimonsky and Bichuette 2019, Vitali-di Castri 1967)

*Neocheiridium* Beier, 1932 is the third largest genus in the family Cheiridiidae. The genus was first described by Max Beier in his monograph on the world pseudoscorpion fauna, with *Cheiridium corticum* Balzan, 1890 from Paraguay as type species (Beier 1932, Balzan 1890). Beier (1959) added one more species from Argentina and Vitali-di Castri (1962) described two new species from Chile. Beier (1978) then added a fifth species from Galapagos Islands. Subsequently, Mahnert (1982) described two Afrotropical species, making *Neocheiridium* a Gondwanan taxon, rather than a strictly Neotropical one and presented a key to the known species. Then Mahnert and Aguiar (1986) redescribed the type species, added two more Neotropical species (including *N. strinatii* from Caribbean islands) and amended the generic diagnosis (see Fig. 1 for the currently known American records).

No North American *Neocheiridium* species are hitherto known (Chamberlin 1924, Hoff 1958, Harvey 2013). Altogether, nine *Neocheiridium* species are known from the Neotropical and Afrotropical regions. In addition, an undescribed species of the genus has been reported from Boatswainbird Island (near Ascension Island) (Rowlands 2001) and there are unpublished records of one species from the Seychelles islands and one from Australia (Mark Harvey, *pers. comm*.).

The generic boundaries in the family Cheiridiidae may need revision (Mahnert 2014, Mahnert and Aguiar 1986, Judson 2000), but as currently understood, the genus *Neocheiridium* is characterised by subtriangular granular carapax with two small eyes, one procurved furrow and "shoulders", abdomen with only 10 (divided) tergites visible from above, rallum with four blades, the distalmost of which is expanded, palp chelal fingers shorter than hand, movable finger with only one trichobothrium, femur and patella of legs fused with suture between them hardly visible and usually body and pedipalp setae curved and covered with cerotegument (Beier 1932, Mahnert 1982, Mahnert 2014, Mahnert and Aguiar 1986).

A new species of *Neocheiridium* is described below, based on specimens of both sexes from the Ohio State University Acarology collection, collected from South Carolina, USA. The finding locality (Hilton Head Island) is a low island near the South Carolina continental coast, with a humid subtropical climate (Köppen classification Cfa), having humid, warm summers and cool and rainy winters. The island has very sandy soil that does not hold water well, drying quickly without percipitation. It is a densely populated area with little natural vegetation remaining.

## Materials and methods

All specimens were extracted from a soil and debris sample using standard Tullgren funnels. The material was kept in 70% ethanol.

Specimens were studied using a Leica S8AP0 dissecting microscope and a Leica DM6000B compound microscope. Line-drawings were prepared using a U-DA drawing tube attached to an Olympus CX31 compound microscope. The images were generated by combining stacks of images with different focal planes using software LAS V.4.5.0.

One specimen (female paratype) was cleared in 10% KOH and mounted on to a slide (in Hoyer's medium), the other specimens being studied in cavity slides in glycerol. The terminology used follows Harvey (1992). All measurements are given in millimetres. The measurements for female are given for holotype, followed by the female paratype in parentheses.

### Abbreviations

The following abbreviations are used for pedipalp trichobothria: t - terminal exterior on movable finger, et - exterior terminal on fixed finger, est - exterior subterminal on fixed finger, esb - exterior subbasal on fixed finger, eb - exterior basal on fixed finger, it - interior terminal on fixed finger, isb - interior subbasal on fixed finger, ib - interior basal on fixed finger.

## Taxon treatments

### Neocheiridium gullahorum

Sammet sp. n.

B18A1CDB-7DF8-529A-B7E6-8C59680B6878

urn:lsid:zoobank.org:act:02840F97-D62A-404C-9724-6831E2D1C6F9

#### Materials

- **Type status:** Holotype. **Occurrence:** catalogNumber: OSAL 0128942; recordedBy: Hans Klompen; sex: female; preparations: in ethanol; **Location:** higherGeography: USA, South Carolina, Beaufort Co.; island: Hilton Head Island; municipality: Sea Pines; verbatimLatitude: 32°08'00"N; verbatimLongitude: 80°47'40"W; decimalLatitude: 32.1332; decimalLongitude: -80.7945; **Event:** samplingProtocol: Tullgren funnel; year: 2004; month: 12; day: 29; habitat: litter and soil in hollow tree; **Record Level:** type: PhysicalObject; institutionID: Ohio State University, Museum of Biological Diversity; institutionCode: OSU; basisOfRecord: PreservedSpecimen
- **Type status:** Paratype. **Occurrence:** catalogNumber: OSAL 0128944; recordedBy: Hans Klompen; sex: male; preparations: in ethanol; **Location:** higherGeography: USA, South Carolina, Beaufort Co.; island: Hilton Head Island; municipality: Sea Pines; verbatimLatitude: 32°08'00"N; verbatimLongitude: 80°47'40"W; decimalLatitude: 32.1332; decimalLongitude: -80.7945; **Event:** samplingProtocol: Tullgren funnel; year: 2004; month: 12; day: 29; habitat: litter and soil in hollow tree; **Record Level:** type: PhysicalObject; institutionID: Ohio State University, Museum of Biological Diversity; institutionCode: OSU; basisOfRecord: PreservedSpecimen
- **Type status:** Paratype. **Occurrence:** catalogNumber: IZBE0310757; recordedBy: Hans Klompen; sex: female; preparations: mounted to slide; **Location:** higherGeography: USA, South Carolina, Beaufort Co.; island: Hilton Head Island; locality: Sea Pines; verbatimLatitude: 32°08'00"N; verbatimLongitude: 80°47'40"W; decimalLatitude: 32.1332; decimalLongitude: -80.7945; **Event:** samplingProtocol: Tullgren funnel; year: 2004; month: 12; day: 29; habitat: litter and soil in hollow tree; **Record Level:** type: PhysicalObject; institutionID: Estonian University of Life Sciences, Entomological Collection; collectionCode: IZBE; basisOfRecord: PreservedSpecimen

#### Description

**Diagnosis**: *Neocheiridium* species with sharp triangular teeth on the distal end of the palpal chela, becoming trapezoidal and gradually flatter towards the proximal end, metazonal depression open to posterior margin, 7 trichobothria on the fixed pedipalp finger and 1 on the movable finger. Palpal chelae length-width ratio with pedicel 2.4-2.6. Body length at least 1 mm, no more than 7 setae on the half-tergites, 4 teeth on the cheliceral fixed finger, female galea without apical branches, tubercles on tergites I-III similar to tubercles of other tergites, two pairs of setae between eyes and cucullus.

**Figure 2.**
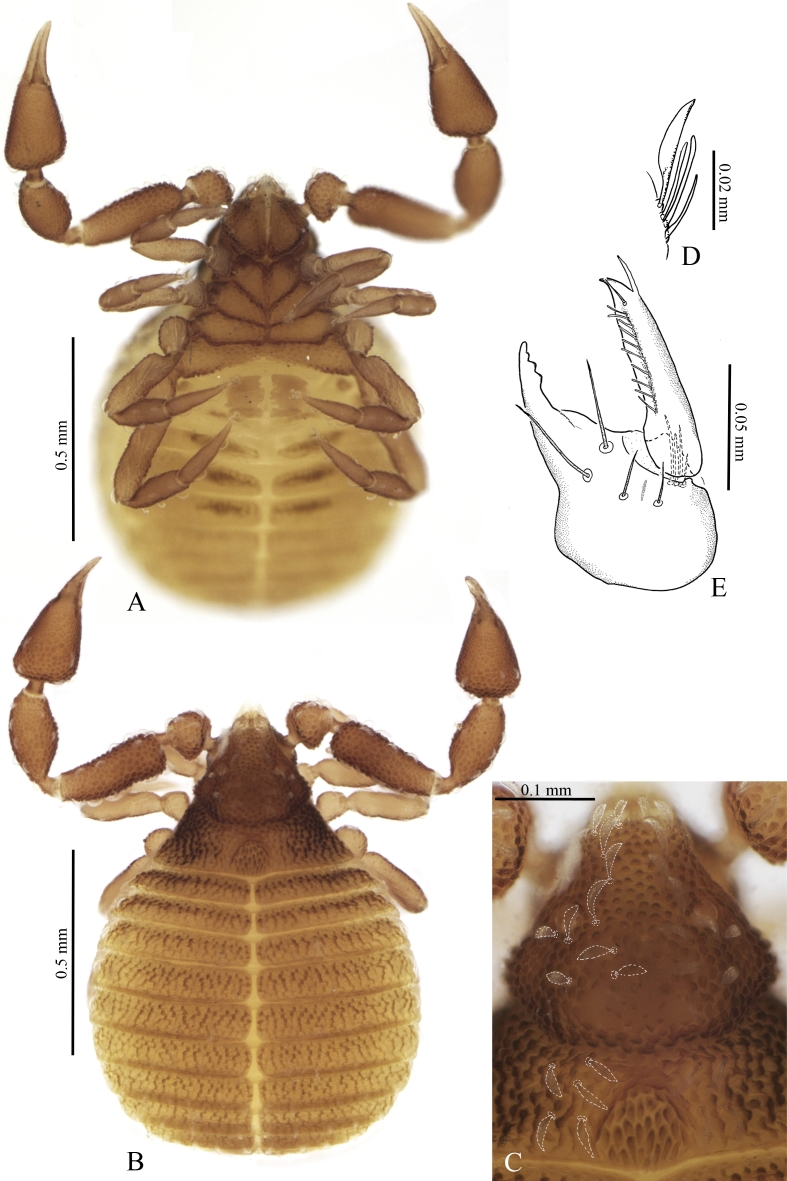
*Neocheiridium gullahorum* n. sp. female **A.** habitus, ventral view (holotype); **B.** habitus, dorsal view (female paratype); **C.** carapax dorsal (holotype); **D.** rallum (female paratype); **E.** chelicera (female paratype).

**Female** (holotype and 1 paratype). Body length 1.12 (1.21) mm (Fig. 2 A andB). Carapax wider than long, 0.36 (0.37) × 0.5 (0.54) mm, dark chestnut brown, with small very weakly bilobed cucullus, two small eyes, well-developed procurved furrow and "shoulders" (Fig. 2 A). Integument tuberculate, the tubercles irregularly polygonal to star-shaped, often longitudinally connected. Metazonal depression parabolic (triangular), open to posterior margin, with longitudinally fusiform granulation, no lateral depressions, posterior margin of the carapax weakly convex, almost angular, with sparse small tubercles. Most setae with cerotegument, giving them leaf-like appearance. Two pairs of setae on the anterior margin of carapax (cucullus), followed by 2 pairs of setae before the eyes and one pair between the eyes. Position of setae in the posterior part of carapax more irregular, total number of setae 19-20 (Fig. 2C).

Abdomen with 10 tergites visible from above (Fig. 2B). I (half)tergites with 2-3 setae in relatively medial position, II tergites with 4 setae, III tergites with 6 setae, IV-VI tergites with 7-8 setae, tergites VII with 6 setae, VIII tergites with 6 setae, IX tergites with 5 setae, X tergites with 3. Setae on carapax and marginal region of abdomen leaf-like and strongly curved, setae on the medial region of tergites and sternites leaf-like and weakly curved. Tubercles on tergites irregularly polygonal and often forming longitudinal to diagonal rows of 2-3 tubercles.

**Figure 3.**
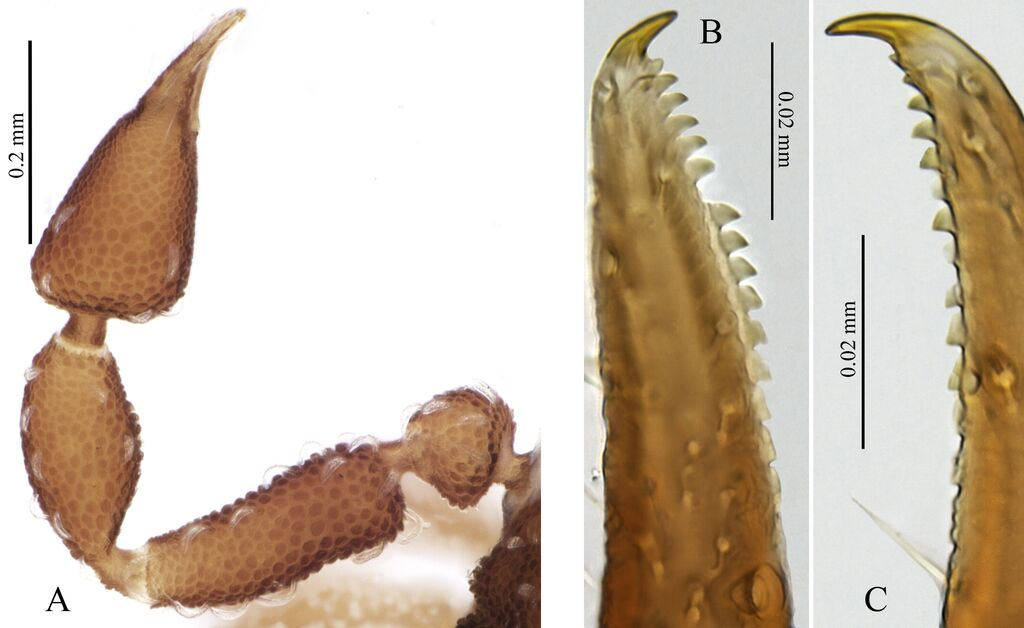
*Neocheiridium gullahorum* n. sp pedipalp (female holotype) **A.** dorsal view; **B.** lateral view of the distal part of the fixed finger; **C.** lateral view of the distal part of the movable finger.

Pedipalps with irregular granulation (the largest tubercles more than 3× larger than the smallest), femur 0.25 (0,26) / 0.09 (0.1), patella 0.22 (0.23) / 0.11 (0.11) (Fig. 3 A). Palpal chelal length 0.32 (0.37), finger 0.16 (0.13). Palpal fixed finger distal teeth longer and conical-recurved, from the 7-8th teeth onwards trapezoidal and gradually lower, 17 on fixed finger (plus one small distal lateral tooth) (Fig. 3 B). Fourteen teeth on the movable finger, distally two small forward directed teeth, followed by larger triangular teeth that become more recurved and lower basally (Fig. 3 C). Fixed finger with 4 external and 3 internal trichobothria, movable finger with 1 external trichobothrium.

**Figure 4.**
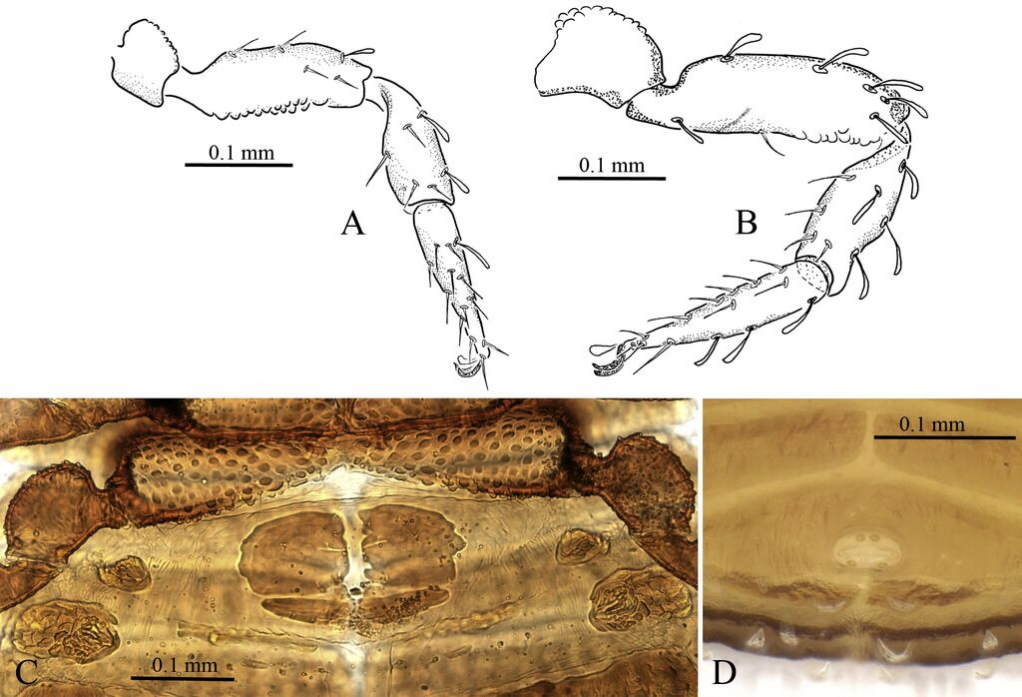
*Neocheiridium gullahorum* n. sp **A.** leg I; **B.** leg IV (female paratype); **C.** genital area (female paratype); **D.** sternite XI (holotype).

Coxae tuberculate (round regularly spaced tubercules), coxa I with sparse tubercules, more concentrated on edges, bearing 4 setae, other leg coxae and palpal coxae more densely tuberculate (Fig. 2 A, Fig. 2Fig. 4C), manducatory process with 3 setae, coxa II with 3 setae, III with 3 setae (?), IV with 6 setae and medially fused. Anus surrounded by two pairs of setae (Fig. 4 D) Leg I: femur 0.16 (0.19) / 0.06 (0.07), tibia 0.14 (0.16) / 0.05 (0.06), tarsus 0.15 (0.16) / 0.04 (0.04) (Fig. 4 B). Leg IV: femur 0.26 (0.27) / 0.083 (0.095), tibia 0.16 (0.17) / 0.055 (0.58), tarsus 0.15 (0.16) / 0.04 (0.45), suture between femur and patella barely visible (Fig. 4 B).

Anterior genital operculum with two irregularly rectangular chitinised plates and medial subparallel lyrifissures, with 6-8 small setae, broadly fused to the next sternite. Posterior operculum with two elongate sclerotised plates bearing 3 setae and two parallel lyrifissures (Fig. 4C).

**Figure 5.**
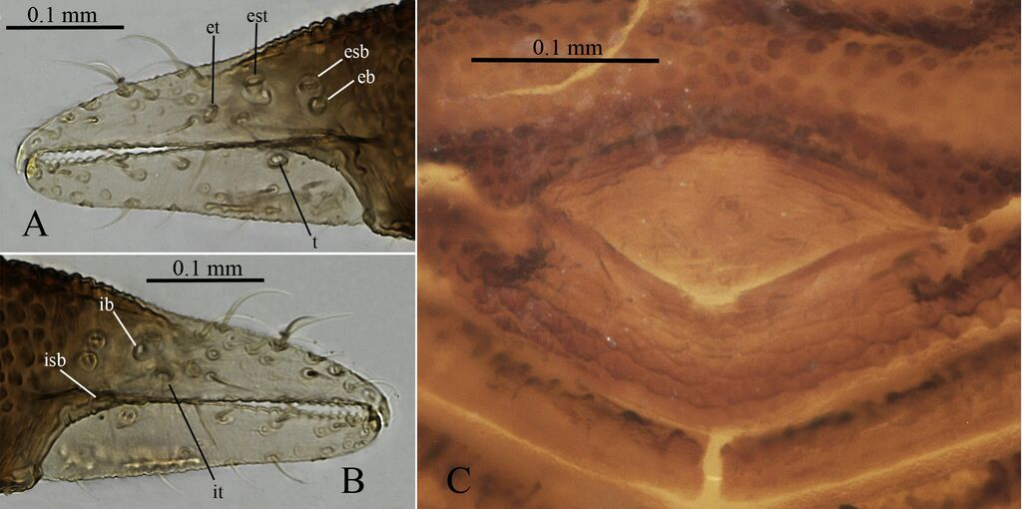
*Neocheiridium gullahorum* n. sp **A.** Pedipalp chela, external lateral view (male paratype); **B.** pedipalp chela, internal lateral view (male paratype); **C.** external genitalia (male paratype).

**Male** (allotype): with similar body proportions, but smaller body length (without chelicerae) ~1.1 mm, carapax 0.33 × 0.49 mm. Body surface sculpture similar to female. Half-tergites I with 2 setae, II - III 4 setae, IV-VII 7 setae, VIII 6 setae, IX 5 setae, X 4 setae. Palpal trochanter 0.08 × 0.09 mm, femur 0.29 × 0.09, patella 0.20 × 0.10, chela 0.34 × 0.14 (length with pedicel 0.37), fingers 0.16. Trichobothria in the same positions as for female, but *isb* a bit lower (Fig. 5A and B). Genital operculum with 12 setae and a central group of 10 shorter setae, posterior operculum with a small medial notch (Fig. 5C).

#### Etymology

The specific name derives from the word „gullah“ used to describe the people and traditional culture of the type locality, with genitive plural ending –*orum*.

## Identification Keys

### Key to the described species of *Neocheiridium* (partially based on Mahnert 1982)

**Table:** 

1	Fixed pedipalp finger with 2 external trichobothria	2
–	Fixed pedipalp finger with 3-4 external trichobothria	3
2	Pedipalp relatively slender, femur 5.2-5.6 times longer than wide (length 0.45-0.48), patella 3.6-3.7 times longer than wide, chela 3.4-3-5 times longer than wide (Argentina).	*N. tenuisetosum* Beier, 1959
–	Pedipalp stouter, femur 3.9-4.1 times longer than wide (length 0.37-0.39), patella 3.1 times longer than wide (length 3.1), chela 2.8 times longer than wide (Chile)	*N. beieri* Vitali-di Castri, 1962
3	Pedipalp relatively slender, femur at least 3.8 times longer than wide; larger species with palp femur at least 0.34 mm.	4
–	Pedipalp relatively plump, femur not more than 3.2 times longer than wide; smaller species, with palp femur not over 0.29 mm.	5
4	Trichobothrium *isb* clearly closer to *ib* than to *ist.* Setae of carapax and tergites short and inconspicuous. Palp femur 5.5 times longer than wide (length 0.34 mm), patella 2.7 times longer than wide (0.29 mm), chela with pedicel 3.06 times longer than wide (Chile)	*N. chilense* Vitali-di Castri, 1962
–	Trichobothrium *isb* halfway between *ib* and *ist.* Setae of carapax and tergites longer and club-shaped. Palp femur 3.9 times longer than wide (length 0.35 mm), patella 2.7 times longer than wide (0.30 mm), chela with pedicel 2.7 times longer than wide (Paraguay, Argentina)	*N. corticum* (Balzan, 1887)
5	Very small species, palp femur 0.17-0.18, 2.4 x longer than wide (Kenya)	*N. pusillum* Mahnert, 1982
–	Larger species with palp femur at least 0.20, at least 2.6 x longer than wide	6
6	Metazonal depression round or oval, open to posterior margin of carapax or not, distal pedipalp teeth triangular	7
–	Metazonal depression subtriangular or parabolic, wide open to posterior margin of carapax, distal teeth of pedipalp fixed finger slender, recurved-conical	9
7	Metazonal depression open to posterior margin of carapax, 3-4 external trichobothria on the fixed pedipalp finger (Galapagos Islands)	*N. galapagoense* Beier, 1978
–	Metazonal depression barely touching posterior margin of carapax, always 4 external trichobothria on the fixed pedipalp finger	8
8	Smaller species, palp femur 0.2-0.21, carapax lighter than pedipalps (Curaçao and Aruba).	*N. strinatii* Mahnert & Aguiar, 1986
–	Larger species, palp femur 0.26-0.29, carapax and pedipalps of the same colour (Kenya).	*N. africanum* Mahnert, 1982
9	Rows of tubercles similar on all tergites. Larger species, body length 1.06-1.21 mm, palpal femur 0.25-0.29, chela with pedicel 2.4-2.6 times longer than wide (South-Eastern United States).	*N. gullahorum* n. sp
–	With more strongly sclerotised rows of tubercles on tergites I-III. Smaller species, body length 0.76-0.97 mm, palpal femur 0.23-0.26, chela with pedicel 2.8-2.9 times longer than wide (Brazil)	*N. triangulare* Mahnert & Aguiar, 1986

## Discussion

The new species seems to be related to *Neocheiridium triangulare* Mahnert & Aguiar, 1986, described from Rio Urubu, Brazil and may be *Neocheiridium galapagoense* Beier, 1978 from the Galapagos Islands. These species share the sharp triangular teeth on the distal end of the palpal chela, becoming trapezoidal and gradually flatter towards the proximal end, the metazonal depression, open to posterior margin and 7 trichobothria on the fixed pedipalp finger and 1 on the movable finger. *N. triangulare* and *N. galapagoense* can be separated from the new species by having more slender palpal chelae (length-width ratio with pedicel 2.8-2.9 and 2.5-2.8, respectively, versus 2.4-2.6 in *N. gullahorum*) and smaller size (body length 0.76-0.97 and 0.90-0.95, respectively, versus 1.06-1.21 in *N. gullahorum*) (Mahnert and Aguiar 1986, Mahnert 2014). It also differs from *N. galapagoense* by having no more than 7 setae on the half-tergites (8-10 in *N. galapagoense*), more slender palpal distal teeth and 4 teeth on the cheliceral fixed finger (2 for *N. galapagoense*). Differences with *N. triangulare* include the lack af apical branches of the female galea, the lack of more strongly sclerotised rows of tubercles on tergites I-III, two pairs of setae between eyes and cucullus, the structure of female external genitalia and smaller number and shape of teeth on pedipalps.

Nothing is known of the ecology of the new species. However, considering its small known range and habitat degradation in the type locality due to recent real estate development there, it may be a vulnerable or endangered species.

## Supplementary Material

XML Treatment for Neocheiridium gullahorum
